# Ecotoxicity of *o*-Chlorobenzylidene Malononitrile (CBM) and Toxicological Risk Assessment for SCLP Biological Cultures (*Saccharomyces* sp., *Chlorella* sp., *Lactobacillus* sp., *Paramecium* sp.)

**DOI:** 10.3390/toxics11030285

**Published:** 2023-03-20

**Authors:** Viorel Gheorghe, Catalina Gabriela Gheorghe, Andreea Bondarev, Raluca Somoghi

**Affiliations:** 1 Oil-Gas University of Ploiesti, 39 B-dul Bucuresti, 100520 Ploiesti, Romania; 2 The National Institute for Research & Development in Chemistry and Petrochemistry, 202, Spl. Independentei, 060021 Bucharest, Romania

**Keywords:** CS gas, *o*-chlorobenzylidene manolonitrile, ecotoxicity, EC_50_, TEM, OD, CFU/mL

## Abstract

Toxic substances used as chemical weapons present a number of particularities that affect the surrounding environment, having a wide range of action by disrupting the ecological balance: they may infect soil or air, or form aerosols through smoke or toxic fog. Such substances can have a long duration of action, from minutes to weeks, which is why they are used in military attacks. This study evaluated the toxicological character of o-chlorobenzyliden malonitrile (CBM) in order to study the toxicity limit of this substance using microbiological cultures of *Saccharomyces* sp., *Chlorella* sp., *Lactobacillus* sp. and *Paramecium* sp., which were used to determine their rate of growth in the presence of different concentrations of o-chlorobenzyliden malonitrile and their ability to respond to this toxic stimulus.

## 1. Introduction

Toxins are chemicals that cause problems in the functionality of the plant or animal organism, depending on the intensity of the action in relation to temperature, pH of the environment, amount of oxygen in the aquatic environment, or conductivity of the solution according to dissolved salts in cases of aquatic contamination. Highlighting the presence of a toxic substance in water and studying its effects on target organisms can be performed by tracking organisms’ behavior and individual resistance [1].

Toxic substances used as chemical weapons present a number of particularities that affect the surrounding environment, having a wide range of action by disrupting the ecological balance: they may infect soil or air, or form aerosols through smoke or toxic fog. Such substances can have a long duration of action, from minutes to weeks, which is why they are used in military attacks. An attack with toxic substances may lead to temporary irritation of the eyes, damage to the airways leading to serious illness, or death.

Aerosols, whose stability is affected by Brownian motion and gravitational deposition, like any colloidal system, are unstable, easily changing their properties, as is the case with irritant tear substances used in the case of dispersal of groups of aggressors or their evacuation from certain areas.

Some toxic substances mainly affect neurons, leading to their deterioration or, when intoxication is serious, their death. The use of CBM in war is prohibited under the terms of the Chemical Weapons Convention (only military use).

A common conception of a chemical weapon (CW) is a toxic chemical contained in a delivery system such as a bomb or artillery shell. While technically correct, a definition based on this conception would only cover a small portion of the range of things the Chemical Weapons Convention (CWC) prohibits as chemical weapons [2].

Explosives are substances that, under the influence of external actions, can undergo rapid chemical transformation, accompanied by a sudden release of heat and the formation of highly heated gases. Shock waves, projectiles, gases, metal particles, powder particles, smoke and flame act specifically when passing through substance in an aerosol state; the specific surface of the substance increases greatly, leading to the acceleration of chemical processes that under normal conditions occur very slowly [3].

Chlorobenzylidene malononitrile (CS) is a compound frequently used in military operations as well as during military personnel training exercises. CS is usually mixed with a pyrotechnic compound for dispersion in grenades or canisters in the form of fine particles that form a characteristic smoke; these are available in either individual containers or large bombs, or can be dispersed using a portable aerosolizer.

The toxicity and degree of danger is also due to the solvents in which the CS powder is dissolved, such as alcohol, ether, carbon sulfide and methylchloroform, which generate breathing difficulties due to the formation of very irritating chlorine atoms and hydrochloric acid when they come into contact with water from mucous membranes. CS is an alkylating agent that targets sulfhydryl groups. In addition, there is some controversy surrounding the production of cyanide molecules at the tissue level with exposure to high concentrations of CS. Exposure can have very serious repercussions if the dispersion is carried out in closed spaces or near the blasts of grenades or other gas spraying devices.

Based on animal studies, a concentration of 25,000–150,000 mg·m^−3^·min^−1^ or 200 mg·kg^−1^ body mass is generally believed to be the lethal dose. It has been determined that a grenade can generate a concentration of 2000–5000 mg·m^−3^ in the blast zone. Due to the poor water solubility of CS, it is difficult to decontaminate and CS left on floors or furniture could become aerosolized again. In addition to the non-persistent form, 2-chlorobenzylidene malononitrile (CS), two hydrophobic variations, CS1 and CS2, were created.

CS1 is a micronized powder containing 5% hydrophobic silica aerogel that can persist for up to two weeks under normal weather conditions, and CS2 is a microencapsulated siliconized form of CS1 with long persistence, degradation resistance, and floatability on the water. This could restrict or prohibit the use of water for the population. Heat during the summer vaporizes CS for dispersion, which thus condenses and forms aerosols, generating a toxic cloud. CS is very rapidly absorbed from the respiratory tract, and the half-life of CS and its main metabolic products is very short. CS decomposition follows first-order kinetics and spontaneously hydrolyzes to malononitrile, which is converted to cyanide in animal tissues.

Metabolically, CS undergoes conversion to 2-chlorobenzyl malononitrile (CSH_2_), 2-chlorobenzaldehyde (o-CB), 2-chlorohippuric acid and thiocyanate. CS and its metabolites can be detected in blood after inhalation exposure. Following inhalation exposure of CS in rodent and non-rodent animal species, two of its metabolites, 2-chlorobenzaldehyde and 2-chlorobenzyl malononitrile, were detected in their blood.

According to the REACH Regulation protocol, acute toxicity refers to the effect of an intense, short-lived exposure of organisms to the action of the toxic compound. The usual duration of acute exposure is generally 96 h (Sprague, 1969) or shorter. A previous work [4] defines, for example, acute oral toxicity by adverse effects occurring after administration of one or more doses of the substance over a period of time; thus, the effects of the toxins can be quantified (e.g., DL_50_/CL_50_—lethal dose/lethal concentration that induces the death of 50% of the tested individuals).

This study aims to evaluate toxicological character in order to study the toxicity limit of some chemicals on microbiological cultures that can be used in experimental studies in the laboratory. These tests show the cellular growth rate of microorganisms in the presence of the toxic CBM and the percentage of inhibition generated by the substance, analyzed on a biofuel.

The intensity of the toxic action of a chemical depends on the organism on which the tests are performed and the chemical’s tolerability at different concentrations. Chemicals in contact with microorganisms are used by them in the biochemical processes in which they are involved, such as metabolic reactions; organic substances are the source of carbon and energy for the biochemical processes through which they obtain the necessary energy for vital activities.

Microorganisms are able to break certain chemical bonds in the molecules of substances. For example, they can cause C–C bond cleavage by decarboxylating ketones or acids. Through the metabolic processes in cells, they can break double bonds or generate hydrolysis reactions that occur with addition or removal of the N atom through NH_3_. All metabolic processes are actively carried out between the bacterium and the nutrient substrate, which is heavily modified by bacterial enzymes through complex biochemical reactions. Consequently, any substrate that microorganisms reach and that allows their development (including medicines) will eventually suffer more or less significant degradation in relation to the chemical component of the substrate and the species of bacteria contaminated.

The addition or removal of hydrogen may be carried out on pairs of carbon atoms next to each other through the respiratory chain, through special redox systems or through intermediate metabolic systems. Through biological oxidation, microorganisms use cellular energy that can be released into oxidation processes by respiration, using molecular oxygen [5].

The scale of effect quantification also applies to irritant or sensitizing effects, which are categorized as weak, moderate, strong or severe, depending on value. In the case of toxins from aqueous solutions, test results allow for the overall assessment of the toxicity of the analyzed impurity. Thus, CE_b 50_ is the concentration of an impurity in solution corresponding to an inhibition of 50% of test microorganisms at a continuous exposure over a fixed period of time.

## 2. Toxicity Analysis

**Figure 1:**
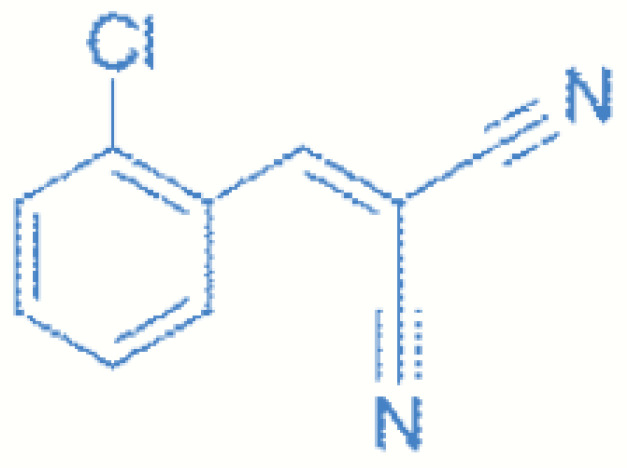
Molecular structure of *o*-chlorobenzyliden malononitrile (CBM).

*o*-chlorobenzyliden malonitrile (CBM) has the chemical formula C_10_H_5_ClN_2_, molecular weight 188.6 g/mol, and water solubility 2.0 × 10^−4^ M. CBM (Figure 1) is an aromatic alkyl nitrile, monosubstituted in the nucleus, with the Cl atom being in the ortho position. It is a derivative of *o*-chlorostyrene [6].

**Figure 2:**
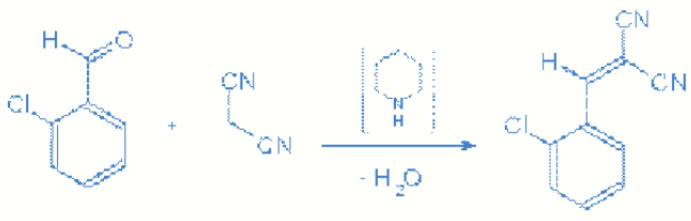
Knoevenagel condensation.

CBM is synthesized through the reaction of 2-chlorobenzaldehyde and malononitrile via Knoevenagel condensation. The reaction is catalyzed by a weak base such as piperidine or pyridine (Figure 2) [7,8].

**Figure 3:**
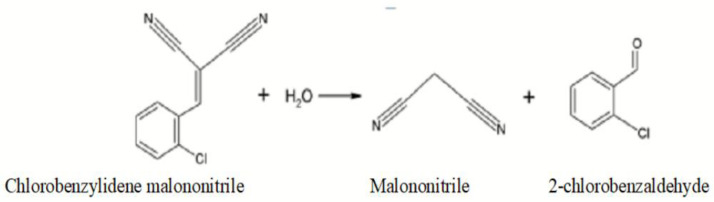
CBM hydrolysis reaction.

In an aqueous neutral environment CBM is relatively stable with respect to hydrolysis. Thus, at a temperature of 30 °C, hydrolysis occurs after 635 min. In an alcoholic environment, the hydrolysis reaction accelerates. For example, in an alcoholic environment with 95% ethanol and 5% water, at 30 °C hydrolysis occurs after 95 min. and at 40 °C hydrolysis occurs after 40 min. Hydrolysis breaks the double ethylene bond via the formation of 2-chlorobenzaldehyde and malonic nitrile (Figure 3) [8,9].

CBM has been widely used as a key ingredient for a number of applications in medical and industrial chemistry and has been used for the synthesis of important medicines such as vitamin B1, aminopterin and amphotericin. It can also be used in the synthesis of lubricants and the manufacture of photosensitive emulsions and tear agents (CS gas, also known as tear gas).

Malononitrile is a chemical compound harmful to the environment that can cause water pollution; it could be completely converted into HCN, a highly toxic product in the metabolism of animal tissues. The presence of CN^−^ anions in the atmosphere can generate a number of serious symptoms, such as eye and skin irritation, dizziness, headaches, dyspnea, convulsions, nausea and vomiting [10].

Acute toxicity is the visible adverse effect induced in an organism within a short period of exposure to a substance [11]. According to the safety data sheet of 2-chlorobenzalmalononitrile, its toxicity for the fish *Pimephales promelas* is an LC_50_ of 0.51 mg/L over 96.0 h, and for *Daphnia magna* (water fleas) and other aquatic invertebrates an EC_50_ of 21.4 mg/L over 24 h [12,13].

Based on the tests performed, the authors determined the toxic agent’s concentration and the dilution rate necessary to preserve this characteristic. This information could be useful for determining the conditions of pollutant (CBM) discharge into an environmental receptor. If discharge is carried out in a river without additional chemical treatment of the contaminated water, the flora and fauna specific to that environment could be affected if the concentration of the pollutant was high. In addition, if the drainage basin is static, the toxic agent’s concentration could gradually accumulate, giving the water a more harmful character. Upon understanding the limits of tolerance for exposure to the toxic substance (CBM) in the aquatic environment, its toxicity could be controlled by supplementing the aquatic flora with specific microorganisms that have a high tolerance to this toxic agent.

## 3. Materials and Methods

### 3.1. Experimental Design

The protocols upon which the research was conducted, using tests on cultures of the micro-organisms *Saccharomyces* sp., *Chlorella* sp., *Lactobacillus* sp., and *Paramecium* sp., obtained the toxicity of *o*-chlorobenzylidene manolonitrile by exposing the cultures in optimal conditions for development in the absence of the toxic agent (control suspension) compared to suspensions of microorganisms in contact with different concentrations of CBM [14].

These cultures of microorganisms were chosen due to the fact that the literature presents aspects of their symbiosis and positive development in adverse environmental conditions, such as under chemical stress. Thus, a symbiotic relationship of microorganisms in the presence of toxic substances showed that they can develop, multiply and biodegrade aromatic toxicants using *Chlorella* sp. algae in relation to *Lactobacillus* sp., or *Chlorella* sp. algae in relation to *Paramecium* sp. ciliate. These microorganisms were tested in the presence of an aromatic inhibitor (phenol) and it was demonstrated that these cultures responded positively, increasing their survival rate together in the toxic environment; an autonomous biosensor integrating the symbiotic association of *Paramecium* sp. and *Chlorella* sp. was successfully developed for real-time monitoring of marine water and evaluation of biotoxicity.

In addition, the microorganism *Lactobacillus* sp. has a high tolerance to chemical stress in symbiosis with the microorganism *Saccharomyces* sp. Numerous tests have revealed the ability of the ciliate *Paramecium* sp. to withstand chemical stress conditions, being able to use *Saccharomyces* sp. yeast as a food source. The food chain could continue by virtue of the fact that the yeast *Saccharomyces* sp. secretes certain growth factors including thiamine and nicotinic acid, which are essential factors for the development of lactobacilli.

In the tests we carried out, the four microorganisms were tested under similar conditions of chemical stress but in separate culture media specific to each individual microorganism. We performed these tests to observe how they react in the presence of CBM, and in subsequent research we will continue experiments and analyze the effect of CBM in the presence of such symbiotic associations.

The working protocol was carried out in two steps. The first step was to prepare the cellular development of the studied microorganisms by developing biomass on selective media, with specific nutrients and under the optimal conditions for each organism. Cell density was quantified by measurements of OD at different wavelengths (400 nm, 500 nm, 600 nm, and 700 nm) to determine the optimal wavelength of light absorption The measurements were carried out using a UV–Vis spectrophotometer. The test was conducted for 28 days; it was possible to observe when the microorganisms reached the exponential growth phase, at which time the cultures were prepared for the second stage of the test.

The second step of the protocol consisted of assessing the toxicity of CBM, prepared as solutions of different concentrations (10–150 µg/mL) that were placed with microorganisms in the logarithmic growth phase of cell development. The tests showed the kinetics of the growth curve for the microorganisms analyzed and the percentage of inhibition that the toxic agent had on the microorganisms. Measurement of the growth curve was conducted by determining the optical density OD at 600 nm using an UV–Vis spectrophotometer, model T85+, PG Instruments.

These tests were able to determine the rate of growth of the microorganisms as an expression of the rate of increase in cell concentration over time, and the microorganisms’ ability to respond to toxic stimuli was analyzed. For each observation period (28 days), the percentage of inhibition expressed as a percentage, the concentration of biomass, the growth rate for each culture studied, and the CE_b 50_ value representing the concentration corresponding to an inhibition of 50% were plotted.

The purpose of the laboratory tests was to study the influence of *o*-chlorobenzylidene malononitrile (CBM) in different concentrations: C1 (10 µg/mL), C2 (25 µg/mL), C3 (50 µg/mL), C4 (75 µg/mL), C5 (100 µg/mL), or C6 (150 µg/mL). Tested solutions were prepared via ultrasonic dispersion using an Ultrasonic SONICA S3-Soltec model. Reactives were weighed using an OHAUS analytical balance model AX224M. A CELESTRON Microscope, model 4434, equipped with a Thoma Marienfeld camera was used for sensitive and rapid image-based detection of microorganism growth. A high-performance multi-parameter WTW Inolab MULTI 9630 IDS with three galvanically isolated measuring channels for pH/mV, ISE, conductivity and oxygen measurement was used for the laboratory tests.

The inhibition measurement was evaluated by reducing the growth or relative growth rate in accordance with SR EN 28692 ISO 8692/2001 13328 ICS 1306030 and SR 13328 ICS 1306040.

Based on the tests performed, the toxic action of the analyzed substance was highlighted within the cellular development process and growth rate of *Saccharomyces* sp. yeast, *Chlorella* sp. green algae, the microorganism *Lactobacillus* sp., and the ciliated organism *Paramecium* sp. [15,16].

These tests were used to calculate EC_b 50_, the mean effective concentration that indicates the concentration of the test substance causing a 50% reduction in growth or growth rate in relation to the control solution [17].

Measurements of cell concentration were performed using the method of counting living cells by comparison with the McFarland nephelometric method, quantifying the measurements by comparison with turbidity standards through establishing correlations with established concentrations of germs.

The stage of culture growth was determined using a UV–Vis spectrophotometer model T85+, PG Instruments, at a wavelength of 400–700 nm, in cells with an optical path of 1 cm. Distilled water with a conductivity less than 5 μS·cm^−1^ was used for dilution [18].

Estimation of microbial production was used both as a general index of cellular developmental metabolic activity and specifically to calculate growth rates, which can lead to considerably different results in measuring growth inhibition. Both shall be used in the interval test to ensure a geometric progression of concentrations that allows for the estimation of EC_b 50_. Growth rate represents increase in cell density over the time unit.

Analysis of biomass growth, in the case of *Chlorella* sp. microalgae, and density of micro-organism biomass, in the case of *Lactobacillus* sp., *Paramecium* sp. and *Saccharomyces* sp. yeast, in the second stage after cell growth was quantified by measuring optical density (OD) using a UV–Vis spectrophotometer at 600 nm.

### 3.2. Microbial Culture Media Preparation

*Saccharomyces* sp. yeast is a single-celled eukaryotic model organism, which is present in a spherical, intermediate form between bacteria and upper fungi. Under strictly anaerobic conditions, some strains become auxotrophs for certain cell components whose synthesis requires molecular oxygen [19].

They have an average size of 4–14 µm. The shape and size of the cell are species characteristics and can be influenced by the physiological state and cultivation conditions. For the development of microorganisms, elements such as nitrogen (N) and phosphorus (P) are required in culture environments. Under aerobic conditions, sugars are assimilated to carbon dioxide and water, thus obtaining the large amounts of energy needed for rapid growth and multiplication.

Yeast cell cultures were grown in a specific medium (1 g yeast extract, 2 g peptone, 2 g dextrose, 2 g NaH_2_PO_4_,). The substances were dissolved in 100 mL of distilled water, and the pH was adjusted to 6.5. Erlenmeyer flasks were placed in a laboratory shaker (ORBITAL Multi-Shaker) at 100 rpm for optimal aeration at 35 °C ± 1 °C. The yeast cell culture was obtained from the Culture Collection of the Petroleum-Gas University of Ploiesti [20].

Microalgae *Chlorella* sp. was cultivated in Erlenmeyer flasks containing 250 cm^3^ of distilled water, MgSO_4_·7H_2_O (0.3 g), KNO_3_ (0.4 g), CaCl_2_ (0.4 g), NaH_2_PO_4_·2H_2_O (0.3 g), FeSO_4_·7H_2_O (0.02 g), NaNO_3_ (0.3 g), and NH_4_Cl (0.2 g); pH was adjusted to 6.5. Erlenmeyer flasks were placed in a laboratory shaker (ORBITAL Multi-Shaker) at 100 rpm for optimal aeration at 35 °C ± 1 °C [21,22].

*Chlorella* sp. is one of the most common freshwater microalgae in aquatic ecosystems. It has the characteristics of high sensitivity to toxic substances, rapid reproduction speed, small volume, wide distribution and ease of obtainment. *Chlorella* is a genus of about thirteen species of single-celled green algae belonging to the division *Chlorophyta*, family *Chlorellaceae*, order *Chlorellales*, class *Trebouxiophyceae*.

The microalgae were cultivated in a bioreactor where the biomass concentration was monitored via UV–Vis spectrophotometry. Cultivation was complete once optical absorbance stopped increasing and the microalgae entered the stationary phase (after ~20 days). Microalgae *Chlorella* sp. was obtained from the Culture Collection of Algae of the Petroleum-Gas University of Ploiesti and adopted as a model organism for experiments [23].

*Lactobacillus* sp. is a microorganism that lives in environments with high carbohydrate content, and is part of the *Firmicutes* inbranch. The culture of the microorganism was obtained from the Culture Collection of the Petroleum-Gas University of Ploiesti and it was adopted as a model organism for experiments. The nutritional solution for the development of *Lactobacillus* sp. was provided by adding cell growth factors in 100 mL of distilled water: 5 g peptone, NaNO_3_ 0.2 g, NH_4_Cl 0.2 g, and Na_3_PO_4_ 5 g. pH was adjusted to 6.0. The culture medium was incubated during the entire test period in a glass-stoppered jug in anaerobic conditions at a temperature of 35 °C ± 1 °C [24].

*Paramecium* sp. belongs to the *Cilliphora* branch, *Ciliata* class, *Holotricha* subclass, *Hymenostomatide* order, *Peniculina* suborder. These are species that live in waters containing decaying nutrients. It is a microscopic organism with a well-defined, flexible and elastic shape. The body of the microorganism has an osmoregulatory function due to two pulsed vacuums. The filling and emptying rate of the pulsed vacuums depends on the temperature of the environment and its salinity. The body responds to certain toxic substances. The multiplication rate of this ciliated organism is asexual multiplication 2–3 times a day; inorganic or organic chemicals can stimulate or slow the division [25].

The product Biolen (biological product containing protozoan microorganisms in an atomized form) was obtained from the Culture Collection of microorganisms of the UPG University of Ploiesti and was adopted as a model organism for experiments. The culture medium was obtained from 10 g Biolen, which was soluble in 100 mL of distilled water to which 25 µg/mL of ampicillin, 35 µg/mL of chloramphenicol, and 25 µg/mL of gentamicin were added to prevent bacterial growth. The nutritional solution for the development of *Paramecium* sp. ciliate was provided by adding cell growth factors: NaNO_3_ 0.3 g, NH_4_Cl 0.2 g, Na_3_PO_4_ 5 g; pH was adjusted to 6.5. Erlenmeyer flasks were placed in a laboratory shaker (ORBITAL Multi-Shaker) at 100 rpm for optimal aeration at 35 °C ± 1 °C [26,27,28].

## 4. Results and Discussion

In the first step, several generations of microbiological cells were cultivated, including young cultures of *Saccharomyces* sp., *Chlorella* sp., *Lactobacillus* sp., and *Paramecium* sp. Microorganisms were raised in culture-specific environments containing growth factors for 28 days by stirring and incubation under continuous white light in a laboratory shaker (ORBITAL Multi Shaker) at 100 rpm for optimal aeration at 35 °C ± 1 °C; light intensity was in the range 60–120 µE∙m^−2^∙s^−1^ [29].

**Figure 4:**
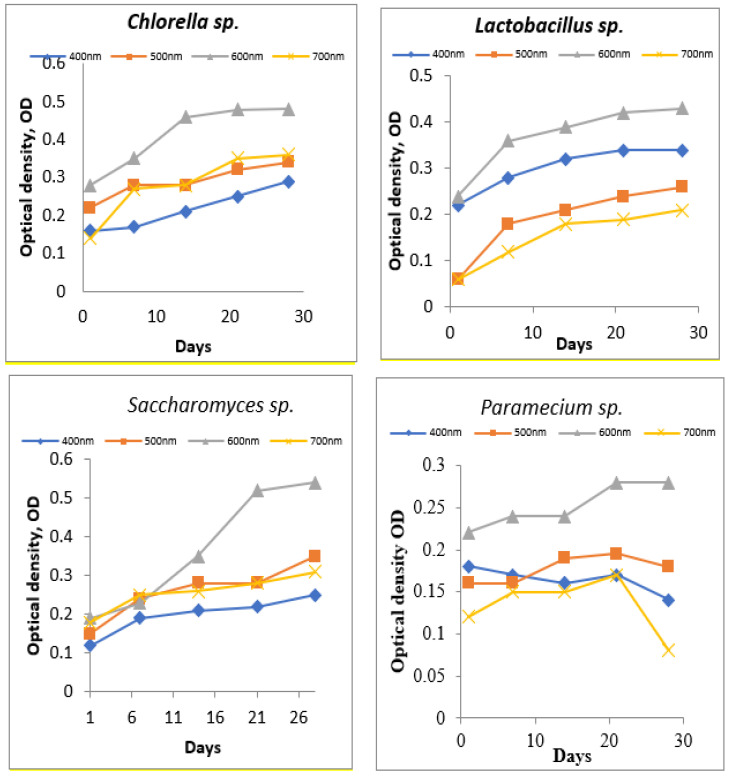
Variation in optical density before treatment.

In this step, the aim was to obtain a young biomass in the exponential growth phase. UV–Vis spectrophotometric measurements were performed periodically to assess cell density. In order to determine the best wavelength where the absorbance is maximum, measurements of OD were made at wavelengths between 400–700 nm (Figure 4).

**Figure 5:**
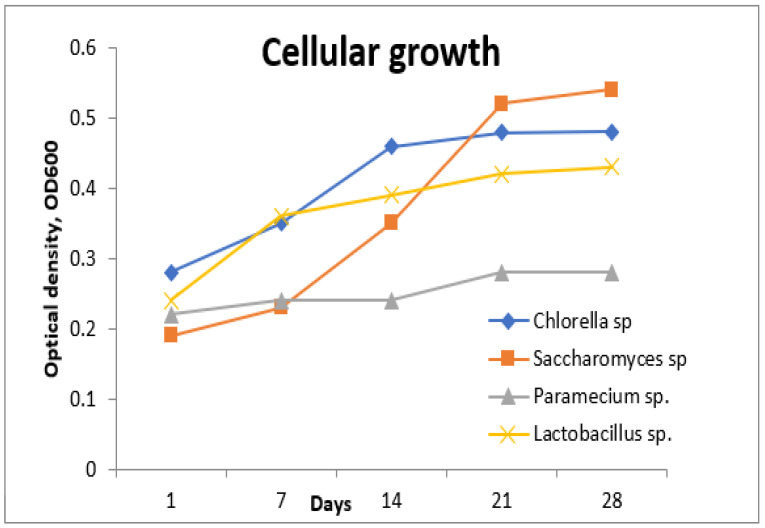
Establishing the exponential growth phases of the tested strains.

Following the tests performed in the first step, it was established that the optimal wavelength for measuring the absorption of the tested strains had the value of 600 nm, due to the conditions provided in the laboratory development environment (Figure 5).

In order to establish the toxic effects of CBM on the strains in the exponential growth phase, it was necessary to study the growth rates of microorganisms in contact with the toxic agent at certain concentrations. For this purpose, it was necessary to obtain solutions of chlorobenzylidene malononitrile (CBM) in different concentrations: C1 (10 µg/mL), C2 (25 µg/mL), C3 (50 µg/mL), C4 (75 µg/mL), C5 (100 µg/mL), and C6 (150 µg/mL), which were prepared by dissolution in water.

For each strain, seven vials were used (bioreactors), in which an aliquot volume of inoculum of 50 mL biological suspension in the exponential growth phase was added, over which a volume of 50 mL specific growth medium was added; in this way, the aim was to obtain a microorganism biomass of about 10^4^ cells/mL. The measurements were taken 24 h after sowing, through microscopic visualization and quantification in the visual field using a Thoma cell counting chamber [30].

Throughout testing, the microorganisms were kept in suspension by mechanical stirring in an orbital shaker to improve gas exchange and reduce pH variation in the test solutions. The strains were kept at a temperature of 35 °C with a photoperiod of 12 h day/night. The bioreactors were inscribed and equipped throughout the testing period with blind stoppers.

Each cell culture of the microorganisms *Saccharomyces* sp., *Chlorella* sp., *Lactobacillus* sp., and *Paramecium* sp. had six vials (bioreactors) and one control vial. At 24 h after the start of testing, chlorobenzylidene malonononitrile (CBM) was added at different concentrations. Thus, in each flask of cell culture of *Saccharomyces* sp., *Chlorella* sp., *Lactobacillus* sp., or *Paramecium* sp. the concentrations were: C1 (10 µg/mL), C2 (25 µg/mL), C3 (50 µg/mL), C4 (75 µg/mL), C5 (100 µg/mL), and C6 (150 µg/mL), while the seventh vial corresponding to each culture medium was considered a control bioreactor. At this point, the start of the test was considered to be the first day in the calculations.

**Figure 6:**
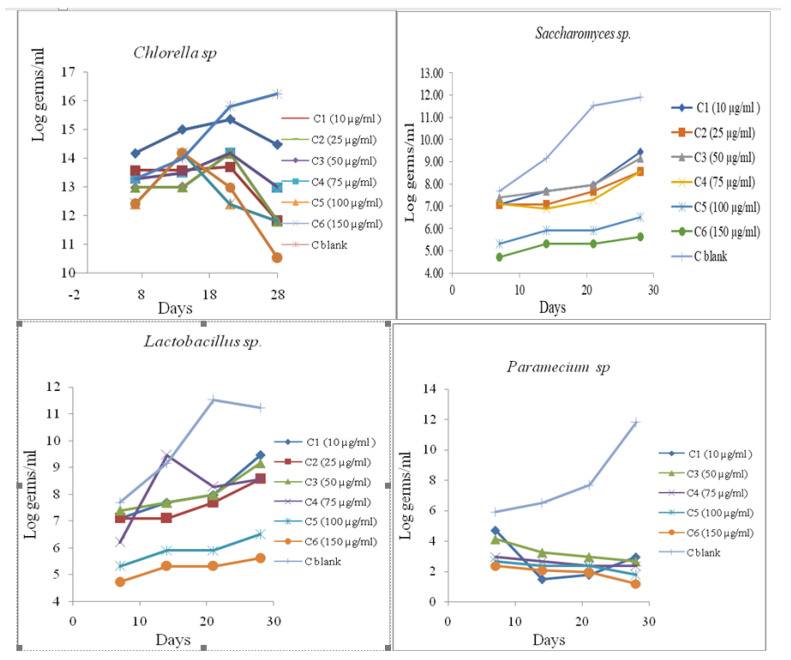
Spectrophotometric measurements of optical density OD_600_.

The second step in our research was to follow the growth of microorganism cultures incubated with the tested substances. Microorganism cells were grown with the chemicals and culture growth was assessed using a spectrophotometric method, by determining OD_600_ at intervals over 28 days. Every seven days, aliquots were taken periodically for culture growth assessment through spectrophotometric measurements of optical density OD_600_ (Figure 6).

The measured cell densities in treated cultures with different concentrations of CBM and control cultures were recorded with the concentrations of the tested substance and the measurement times. Cell growth curves were determined by calculating the average density of cell development during the experiment in the presence of the established concentrations of CBM.

Microbial growth leads to detectable changes in environmental opacity. This could be quantified by measuring optical density with a spectrophotometer at a wavelength of 600 nm. Turbidity does not indicate the viability of microorganisms, and therefore the method does not distinguish between viable and nonviable microorganisms. In order to observe the viability of the tested microbial stem in polluted environments, after each photometering, decimal dilutions (10^−4^) were conducted with saline. The diluted samples were inoculated (1 mL of decimal dilutions of 10^−4^) in a specific nutrient culture medium poured into Petri dishes. The growth of the microbial cultures was investigated via the plate method at specified intervals. The results are presented as log colony-forming units (CFU)/milliliter.

The growth of *Chlorella* sp. was monitored by plate counts pipetted in TSB agar square plates (3% TSB (Tryptic Soy Broth g/l content purified water, Tryptone 17.0 g, Soytone (Peptic Digest of Soybean) 3.0 g, Glucose 2.5 g, Sodium Chloride 5.0 g, Dipotassium Phosphate 2.5 g, pH 7.3 ± 0.2 and 1.5% agar in H_2_O). The plates were incubated at 30 °C and CFU were counted after 48 h.

Viability testing on *Saccharomyces* sp. was monitored by plate counts pipetted in Sabouraud medium (bacto-peptone 10 g, glucose 20 g, agar 20 g, distilled water up to 1000 mL). The plates were incubated at 20°C and CFU were counted after seven days of incubation.

To observe the viability of *Lactobacillus* sp. in polluted environments, counts were conducted of bacterial colonies developed in a nutrient agar culture medium (bacto-peptone 10 g, NaCl 5 g, agar 20 g, meat extract 10 g, distilled water up to 1000 mL) at 45 °C. The diluted samples were inoculated (1 mL of decimal dilutions of 10^−4^) in nutrient agar medium poured into Petri dishes. The samples were scrambled for incorporation and, after cooling, were incubated in thermostat at 30 °C for 48 h.

Viability testing of microorganisms in control environments was followed by testing of the viability of microorganisms in environments with CBM, in comparison with the viability of microorganisms without contaminators. The percentage of viable cells yielded was calculated by treating the control culture growth as 100%.

For the quantification of *Paramecium* sp. ciliate and measurement of the viability of this organism, the number of prokaryotic microorganisms per milliliter of the sample was estimated by microscopic counting on wet preparation and gram-color fixed smear preparation. The estimate of the number of cells was performed on a sample of about 10 images for each concentration of CBM.

For determination of the concentration/effect relationship of CBM, the points indicating inhibition between 0 and 100% were taken into account [31,32].

The areas between the growth curves were calculated according to Equation (1):

(1) $$A=\frac{N_{1}-N_{0}}{2}\;\cdot \;t_{1}\;+\frac{N_{1}+N_{2}-2N_{0}}{2}\;\left(t_{2}-t_{1})+\ldots \ldots +\frac{N_{n-1}+N_{n-2}N_{0}}{2}\;(t_{n}-t_{n-1}\right)$$

where the values are represented by measurements performed at 7, 14, 21 and 28 days; *t* represents time and *N*_1_ represents the number of cells/mL measured at *t*_1_; *N*_0_ represents the number of cells/mL measured at the beginning of the test; *N_n_* represents the number of cells/mL measured at *t*_4_.

**Figure 7:**
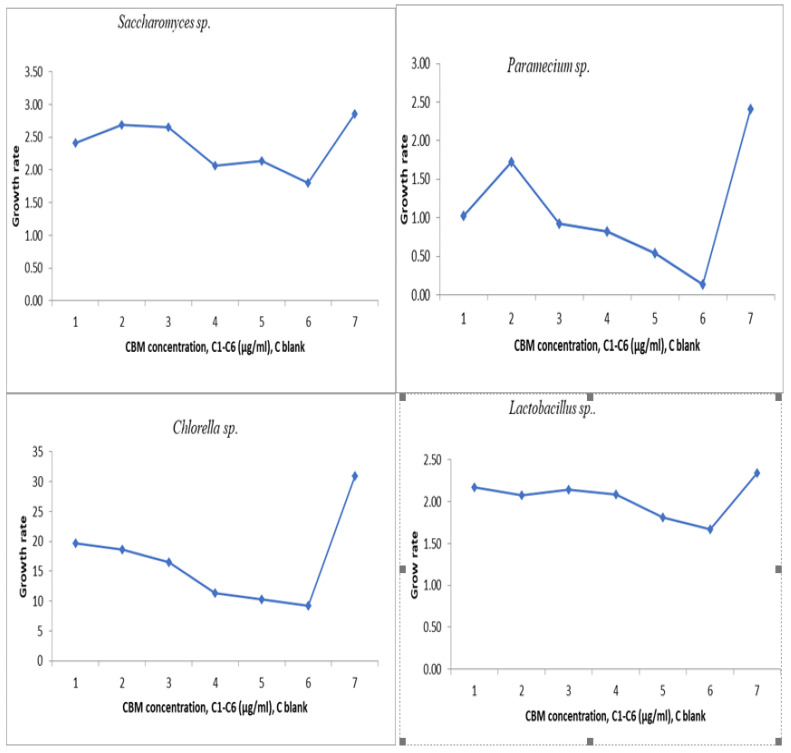
Average growth rate.

The average specific growth rate (*μ*) was calculated as an expression referring to the logarithmic increase in biomass during the exposure period and calculated by Equation (2):

(2) $$\mu =\frac{ln\;N_{n}-lnN_{0}}{t_{1}-t_{n}}$$

where *t*_1_ = is the time at the start of the test; *t*_n_ is the time at the end of the test. The average specific growth rate results from the slope of the regression line of the time-based chart *ln N*. The area under the biomass growth curve was calculated for each culture medium that was tested. The experimental results obtained are presented in Figure 7.

**Figure 8:**
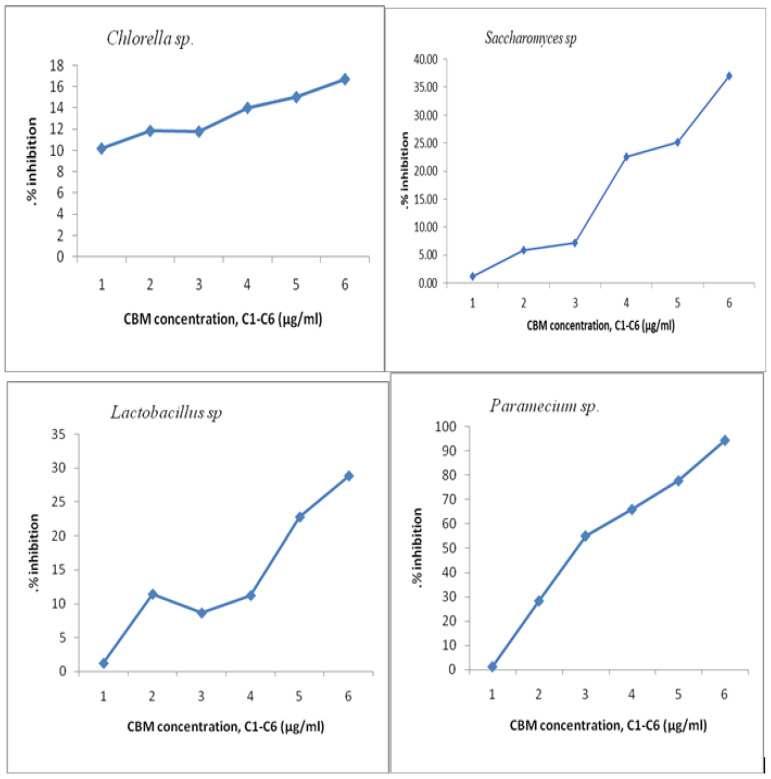
Inhibition of growth rate.

The mean value of A for each concentration and control solution was used to express the percentage of inhibition that each tested concentration had on the analyzed cultures. The percentage of cell growth inhibition at each concentration of the tested substance (I_A_) was calculated according to the following Equation (3):

(3) $$\;I_{A}=\frac{A_{c}-A_{i}}{A_{c}}\;\cdot \;100$$

where *A_c_* represents the mean area of the cell culture control and *A_i_* is the average area of the tested sample of concentration I. The results are presented in Figure 8.

From the graph of the changes in cell density increase in all analyzed samples, it can be seen that the microorganism *Chlorella* sp. had a growth rate evolution (μ) of around 2.5 in contact with the concentrations analyzed. From the graph it can be seen that for the maximum concentration of 150 μg/mL, *Chlorella* sp. was not severely inhibited, and the toxic substance’s concentration did not greatly influence the increase in the final concentration of algae, which was (μ) below 2.5. The maximum percentage of inhibition of *Chlorella* sp. culture was almost 20%, which means that the algae tolerates this substance and the concentrations studied are not very destructive to this microorganism.

The *Saccharomyces* sp. culture was inhibited by more than 40% at the maximum concentration of 150 μg/mL, the cell growth rate at this concentration being almost 2, while at the minimum concentration of 10 μg/mL, inhibition of the culture was not significant, the cell growth being almost 2.5. In the next concentration range, 50 μg/mL to 75 μg/mL, cell mass growth reached a maximum of almost 2.75, with the highest value of cell growth of all studied cultures.

The percentage of inhibition of *Lactobacillus* culture at a maximum concentration of 150 μg/mL was 35%. The minimum concentration of 10 μg/mL inhibited *Lactobacillus* culture to a small extent, while a concentration of 75 μg/mL had an inhibition of almost 15%.

**Figure 9:**
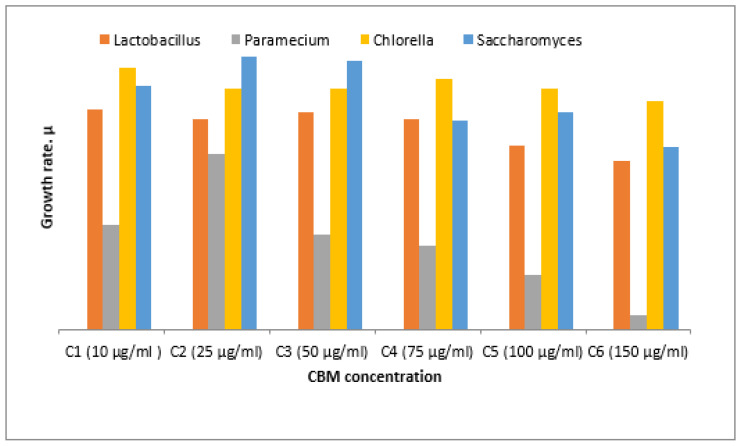
Growth profile of microorganisms.

**Figure 10:**
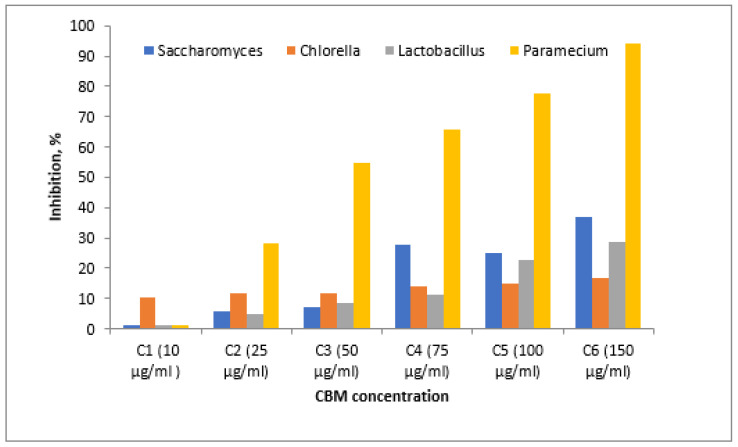
The percentage inhibition of the tested biological cultures.

For *Paramecium* sp., biological mass development had a maximum in the sample with a CBM concentration of 75 μg/mL, where the cell growth rate was 1.7 compared to the lowest growth rate at 150μg/mL, where it had a value of 0.20. The culture of *Paramecium* sp. had maximum inhibition at a CBM concentration of 150 μg/mL, above 90%, proving very sensitive to this substance. A lower concentration of 100 μg/mL inhibited the cellular development of *Paramecium* sp. by 55% (Figure 9 and Figure 10). The percentage inhibition of the tested biological cultures can be seen in Figure 10.

**Figure 11:**
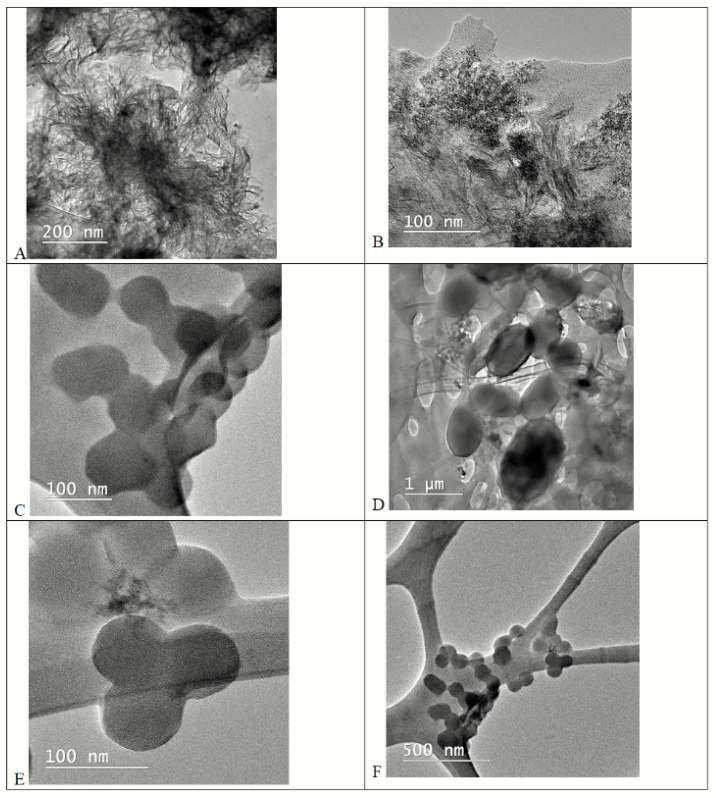
TEM images of *Chlorella* sp. Cellular structure: (**A**,**B**) CBM, 15 days, 150 µg/mL; (**C**,**D**) Blank 15 days; (**E**,**F**) Blank 28 days.

Good development in relation to the other bacterial cultures was shown by the microorganism *Chlorella* sp. Detailed aspects of the zoogleal gelatinous mass that formed around the culture can be observed in Figure 11, due to the growing environment in the presence of the toxic agent at a concentration of 150 μg/mL after 15 days of contact (A, B). Control biomass of the *Chlorella* sp. culture is detailed in points (C, D) after 15 days of growth, and (E, F) for *Chlorella* sp. biomass after 28 days of growth.

**Figure 12:**
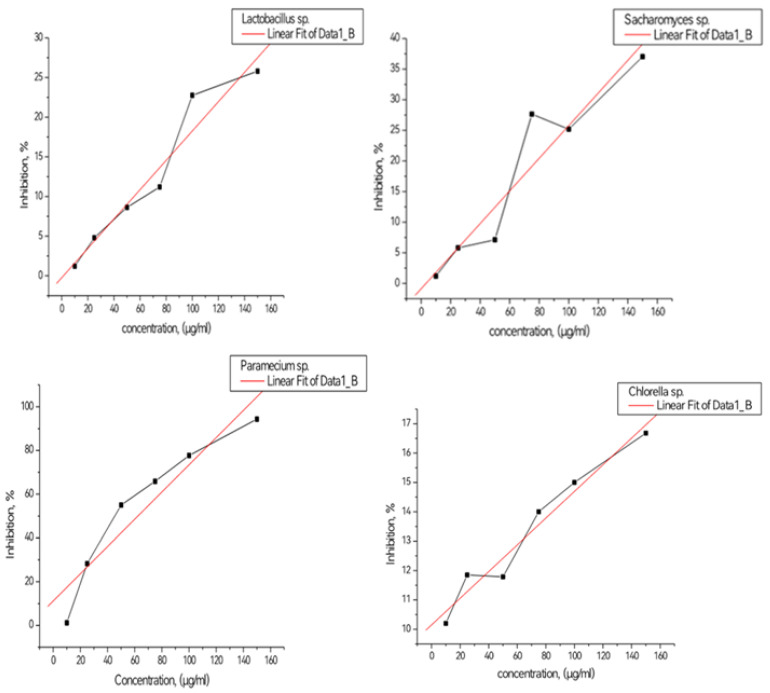
Estimation of EC_b50_.

EC_b50_ value was estimated from the regression line by reading on the graph the concentration equivalent to 50% inhibition. This value is found as the symbol EC_b50_ and represents the inhibition during the corresponding exposure period. The experimental results obtained are presented in Figure 12. EC_b50_ value was determined from the slope of the calibration curve, estimating the concentration corresponding to a 50% inhibition of the tested microorganisms.

From the graph it can be seen that the slope towards the right indicates for *Saccharomyces* sp. culture an inhibition of 50% around 0.25 mg/mL, and for *Chlorella* sp. culture EC_b50_ corresponds to 0.44 mg/mL. *Lactobacillus* sp. culture had EC_b50_ around 0.3 mg/l. In terms of the *Paraecium* sp. culture, EC_b50_ had a value of 50 μg/mL, this culture being the most sensitive to the action of CBM. Our results present evidence that the most resistant cell culture in the presence of CBM was *Chlorella* sp. algae.

**Figure 13:**
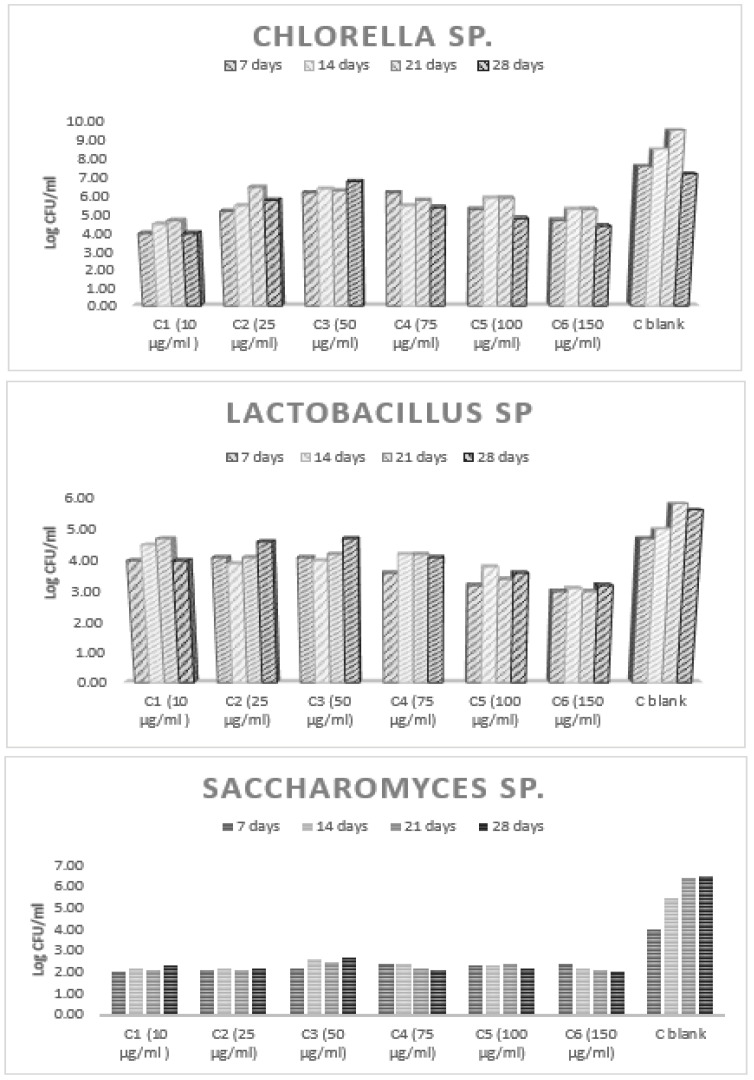
Cellular viability.

The viability efficiency was interpreted taking into account the average of the CFU/mL values and the numbers of viable cells resulting from growth in the specific culture environments (Figure 13).

**Figure 14:**
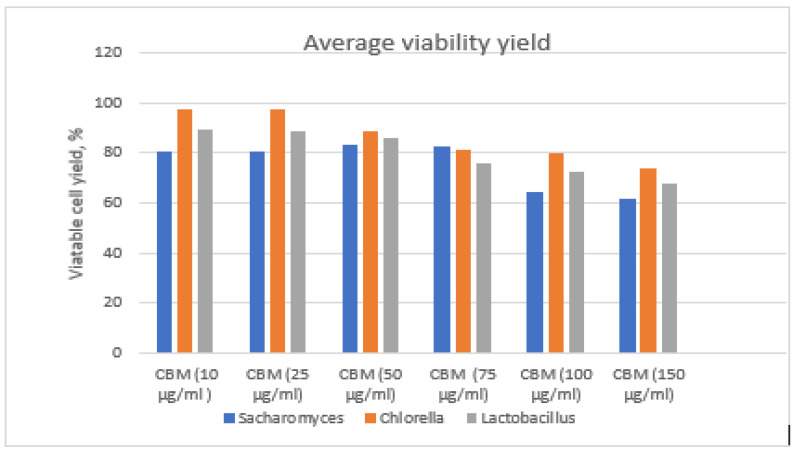
Average viability yield.

For determination of the yield, the ratio of the viability of cells analyzed in the presence of the toxic agent (viable CFU/mL) to the viability of the control cells (CFU/mL, cells developed in non-toxic culture environments) was determined, with the latter considered to be 100% (Figure 14).

*Chlorella* has the highest survival rate in the environment contaminated with CBM and had a rapid response ability to toxic stimuli. The cell viability percentage was above 97.4% for the 10 µg/mL CBM concentration and was 74% for the 150 µg/mL CBM concentration.

*Lactobacillus* had a higher viability rate than *Saccharomyces*, with 89% viable cells found in the treated environment at 10 µg/mL CBM and 68% for the highest concentration, 150 µg/mL CBM.

The yeast *Saccharomyces* sp. had an average pro-center viability of 80.8% for the concentration of 10 µg/mL CBM and 62% for the concentration of 150 µg/mL CBM; the percentage of viability is lower for *Saccharomyces* than *Chlorella*. We assume that this is due to the suppressive effect that the toxic substance generated when it was analyzed, affecting the genetic system of yeasts, decreasing the synthesis capacity of nuclear material, and inhibiting multiplication.

## 5. Conclusions

Studies conclude that *Chlorella* sp., *Lactobacillus* sp., *Saccharomyces* sp., and *Paramecium* sp. can be used in processes for the bioremediation of contaminated ecosystems. The experiments showed that microorganisms are able to utilize CBM to increase biomass, but cell viability in the presence of these contaminators is different for tested microorganism species, which established competing relationships for the substrate. Tests showed that the most resistant cellular strain tested in the presence of CBM is the algae *Chlorella* sp. The concentrations of CBM to which the microorganisms were exposed induce oxidative stress and inhibition of growth; the toxicological response varied according to experiential factors, duration of exposure and exposure concentration. These results could be used to assess and control CBM ecotoxicity in cases of contamination in the natural environment.

As far as basic theoretical research is concerned, in future research we also aim to study the effect of the primary metabolites of CBM that break down via conversion into 2-chlorobenzyl malonononitrile (CSH_2_), 2-chlorobenzaldehyde (O-CB), 2-chlorohippuric acid (C_9_H_8_ClNO_3_) and thiocyanate. CBM metabolites will be studied through experimental testing of the individually developed biological cultures and in synergic ratios to assess their ecotoxicity and their mechanisms of action. In the case of the natural applications of studies on accidental or intentional pollution (used in the case of military instruction or operations with CBM), we aim to improve measurement systems for the concentrations of the main pollutants and their metabolites.

We also aim to establish chemical–biological intervention protocols by researching and quantifying a dose–response relationship between the intensity of chemical indicators and the response of target microorganisms.

## Data Availability

Not Applicable.
